# Complete Genome Sequence of the Complex Carbohydrate-Degrading Marine Bacterium, *Saccharophagus degradans* Strain 2-40^T^

**DOI:** 10.1371/journal.pgen.1000087

**Published:** 2008-05-30

**Authors:** Ronald M. Weiner, Larry E. Taylor, Bernard Henrissat, Loren Hauser, Miriam Land, Pedro M. Coutinho, Corinne Rancurel, Elizabeth H. Saunders, Atkinson G. Longmire, Haitao Zhang, Edward A. Bayer, Harry J. Gilbert, Frank Larimer, Igor B. Zhulin, Nathan A. Ekborg, Raphael Lamed, Paul M. Richardson, Ilya Borovok, Steven Hutcheson

**Affiliations:** 1 Department of Cell Biology and Molecular Genetics, University of Maryland, College Park, Maryland, United States of America; 2 Marine and Estuarine Environmental Sciences Program, University of Maryland, College Park, Maryland, United States of America; 3 Architecture et Fonction des Macromolécules Biologiques, UMR6098, CNRS, Universités Aix-Marseille I & II, Marseille, France; 4 Oak Ridge National Laboratory (ORNL), Life Sciences Division, Oak Ridge, Tennessee, United States of America; 5 Joint Genome Institute, Group B-5 Los Alamos National Laboratory, Los Alamos, New Mexico, United States of America; 6 Department of Biological Chemistry, Weizmann Institute of Science, Rehovot, Israel; 7 Institute for Cell and Molecular Biosciences, University of Newcastle upon Tyne, Newcastle upon Tyne, United Kingdom; 8 Joint Institute for Computational Sciences, University of Tennessee–Oak Ridge National Laboratory, Oak Ridge, Tennessee, United States of America; 9 Department of Molecular Microbiology and Biotechnology, Tel Aviv University, Tel Aviv, Israel; 10 DOE Joint Genome Institute, Production Genomics Facility, Walnut Creek, California, United States of America; Stanford University, United States of America

## Abstract

The marine bacterium *Saccharophagus degradans* strain 2-40 (Sde 2-40) is emerging as a vanguard of a recently discovered group of marine and estuarine bacteria that recycles complex polysaccharides. We report its complete genome sequence, analysis of which identifies an unusually large number of enzymes that degrade >10 complex polysaccharides. Not only is this an extraordinary range of catabolic capability, many of the enzymes exhibit unusual architecture including novel combinations of catalytic and substrate-binding modules. We hypothesize that many of these features are adaptations that facilitate depolymerization of complex polysaccharides in the marine environment. This is the first sequenced genome of a marine bacterium that can degrade plant cell walls, an important component of the carbon cycle that is not well-characterized in the marine environment.

## Author Summary

A segment of the global marine carbon cycle that has been poorly characterized is the mineralization of complex polysaccharides to carbon dioxide, a greenhouse gas. It also remained a mystery whether prokaryotes mineralize plant/algal cell walls and woody material in the oceans via carbohydrase systems and, if so, which organisms are involved. We have analyzed the complete genome sequence of the marine bacterium *Saccharophagus degradans* to better ascertain the potential role of prokaryotes in marine carbon transformation. We discovered that *S. degradans,* which is related to a number of other newly discovered marine strains, has an unprecedented quantity and diversity of carbohydrases, including the first characterized marine cellulose system. In fact, extensive analysis of the *S. degradans* genome sequence and functional followup experiments identified an extensive collection of complete enzyme systems that degrade more than 10 complex polysaccharides. These include agar, alginate, and chitin, altogether representing an extraordinary range of catabolic capability. Genomic analyses further demonstrated that the carbohydrases are unusually modular; sequence comparisons revealed that many of the functional modules were acquired by lateral transfer. These results suggest that the prokaryotic contribution to marine carbon fluxes is substantial and cannot be ignored in predictions of climate change.

## Introduction

Carbon cycle fluxes are critical to understanding global warming equations. Many of the terrestrial prokaryotes that fix CO_2_ have been studied; and many of the microorganisms that degrade approximately 119 pentagrams of carbon per year (PgC/yr) on land have been characterized. Likewise, prokaryotic CO_2_ fixation (i.e. photosynthesis) has been studied in the oceans, with cyanobacteria, e.g. *Synechococcus* and *Procholorcococcus*, found to be major contributors [1],[2]. What has remained a mystery is whether prokaryotes mineralize plant/algal cell walls and woody material in the oceans and, if so, which organisms are responsible. That is, much less is known about how the approximately 97 PgC in complex polysaccharides that are produced each year in the oceans are recycled to CO_2_ [http://science.hq.nasa.gov/oceans/system/carbon.html]. These include complex polysaccharides associated with biofilms, planktonic organisms, algal blooms, shells of benthic invertebrates, and especially higher plant material. Recently, several related bacterial genera that carry out these processes have been discovered [3],[4], either by isolation or metagenomics. A recent wide-ranging metagenomic global expedition revealed that genes related to these taxa are among the most abundant in the oceans [5]. These recently recognized organisms are likely to have a key role in the recycling of marine biomass carbon, thereby enhancing the turnover rates of recalcitrant complex polysaccharides and thus contributing to atmospheric CO_2_ inputs.

*Saccharophagus degradans* strain 2-40^T^ (Sde 2-40; formerly *Microbulbifer degradans* strain 2-40), is the first free-living marine bacterium demonstrated to be capable of degrading cellulosic algae and higher plant material. 16S rDNA analysis shows that Sde 2-40 is a member of the gamma-subclass of the phylum *Proteobacteria*, related to *Microbulbifer hydrolyticus* [3] and to *Teredinibacter sp.,* [4], cellulolytic nitrogen-fixing bacteria that are symbionts of shipworms. The classification of Sde 2-40 has recently been clarified, with its placement in a new genus, *Saccharophagus degradans*, [6] that forms a third genus in this newly emerging *Microbulbifer*/*Teredinibacter/Saccharophagus* group of marine carbohydrate degraders. Of the 20 isolates in this group, the genome of Sde 2-40 is the first to be sequenced.

Sde 2-40 was isolated from decaying salt marsh cord grass, *Spartina alterniflora,* in the Chesapeake Bay watershed [7]. It is a pleomorphic, Gram-negative, aerobic, motile gamma-Proteobacterium, uniquely degrading at least 10 different complex polysaccharides, including agar, chitin, alginic acid, cellulose, β-glucan, laminarin, pectin, pullulan, starch and xylan [8]. These enzymatic capabilities initially suggested that Sde 2-40 has a significant role in the marine carbon cycle: functioning as a “super-degrader” and mediating the breakdown of complex polysaccharides from plants, algae and invertebrates. It represents an important and understudied system.

The Sde 2-40 genome, sequenced to completion and closed (http://genome.jgi-psf.org/finished_microbes/micde/micde.home.html, Accession # CP000282), has 4008 genes in a single replicon consisting of 5.06 Mb (Table 1). The genome annotation reveals that Sde 2-40 is unique in its variety of depolymerases and unusual in its number of open reading frames coding for complex polysaccharide depolymerases. These carbohydrases and related proteins, comprising 10% of the genome, contain extraordinary modularity, interesting architecture and a remarkable proportion of membrane targeting domains. Although such an arrangement is limited to few taxa, the bacteria within these taxa appear to be widely distributed in marine and estuarine waters.

**Table 1:** General features of the *S. degradans* 2-40 genome.

Category	Number	% of Total
DNA, total number of bases	5057531	100.00%
DNA coding number of bases	4385202	86.71%
DNA G+C number of bases	2317668	45.83% 1
DNA scaffolds	1	100.00%
Genes total number^2^	4067	100.00%
Protein coding genes (including pseudogenes)	4017	98.77%
RNA genes	50	1.23%
rRNA genes	6	0.15%
5S rRNA	2	0.05%
16S rRNA	2	0.05%
23S rRNA	2	0.05%
tRNA genes	41	1.01%
Other RNA genes (*rnpB*, *ffs* and *ssrA*)	3	0.08%
Genes with function prediction	2809	69.07%
Genes without function prediction	1208	29.70%
Genes w/o function with similarity	1206	29.65%
Genes w/o function w/o similarity	2	0.05%
Pseudogenes^3^	9	0.22%
Genes assigned to enzymes	403	9.91%
Genes connected to KEGG pathways	404	9.93%
Genes not connected to KEGG pathways	3663	90.07%
Genes in ortholog clusters	3611	88.79%
Genes in paralog clusters	456	11.21%
Genes in COGs^4^	2440	60.00%
Genes in Pfam	2748	67.57%
Genes in InterPro	2953	72.61%
Genes with IMG Terms	327	8.04%
Genes in IMG Pathways	172	4.23%

1 GC percentage shown as count of G's and C's divided by a total number of G's, C's, A's, and T's. This is not necessarily synonymous with the total number of bases.

2 Includes genes encoding proteins, RNA genes and pseudogenes.

3 Pseudogenes may also be counted as protein coding or RNA genes, so is not additive under total gene count.

4 See <http://www.ncbi.nlm.nih.gov/sutils/coxik.cgi?gi=19331>.

## Results/Discussion

### Genome Organization

**Figure 1:**
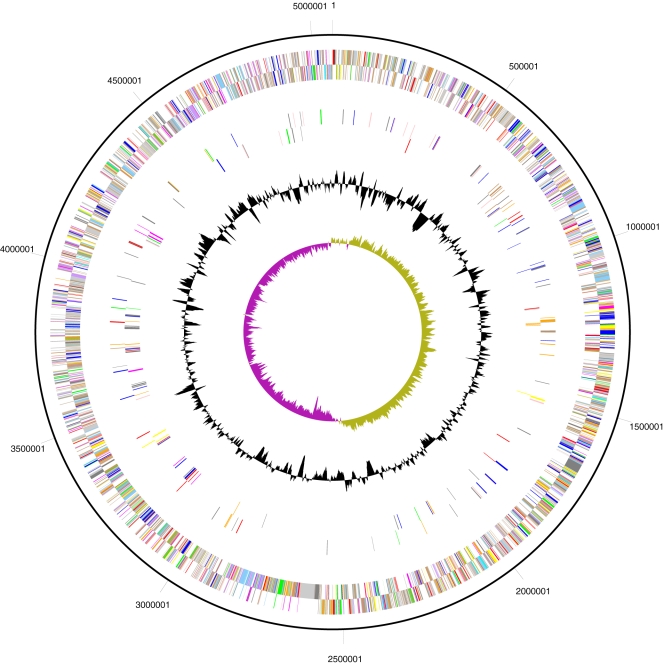
Schematic circular representation of the *S. degradans* 2-40 closed genome sequence. The nucleotide sequence of the 5.06 Mb *Sde*2-40 genome was determined by the United States Department of Energy Joint Genome Institute. Outer ring, sequence address in nucleotides. Next pair of rings, location of the identified 4009 gene models on each coding strand of the genome and predicted function of products: replication and repair (red), energy metabolism (green), carbon and carbohydrate metabolism (blue), lipid metabolism (cyan), transcription (magenta), translation (yellow), amino acid metabolism (orange), metabolism of cofactors and vitamins (pink), purine and pyrimidine metabolism (light red), signal transduction (lavender), cellular processes (sky blue), structural RNA's (pale green) miscellaneous functions (brown), conserved and unique hypothetic proteins (light or dark grey). Next pair of rings, location of predicted or known carbohydrase genes. cellulase (red), chitinase (green), hemicellulase (blue, includes xylanases, arabinofuranases, mixed function glucanases), pectinase (orange), carbohydrate binding module protein (black), alginase (purple), agarase (yellow). Black ring, deviation from the average %( G+C), Inner ring, GC Skew (G−C)/(G+C)

The genome of Sde 2-40 is a single circular chromosome of 5,057,531 bp (the general features of the genome are listed in Table 1, and a detailed map is shown in Figure 1). Nucleotide 1 was assigned at the predicted origin of replication. Overall, the Sde 2-40 genome is 45.8% G+C. A total of 4,008 protein-encoding genes were predicted, averaging 1,094 bp in length, with intergenic regions averaging 166 bp. The open reading frames (ORFs) account for 4,385,202 nucleotides of coding sequence (86.7%). An additional 9 ORFs are classified as pseudogenes (Table 1). Of the 4,008 putative proteins, 3,795 matched a sequence in the NR database with an e-value of <1e-5; of these, 2994 were given a functional assignment based on similarity to a COG group, and 704 were classified as conserved hypothetical proteins. For 2575 of the genes with an identifiable ortholog, the closest homolog was found in a gamma proteobacterium with the largest representation (1057) present in a fluorescent pseudomonad or the closely related *Hahella chejuensis*. Nearly all of these genes appear to function in basic metabolism suggestive of an ancestral relationship; but little synteny was observed with several *Pseudomonas* genomes or *Hahella chejuensis* (data not shown).

In addition to protein-encoding genes, forty one genes for tRNAs and two rRNA gene clusters, located on different strands, were identified (Table 1). Both numbers are atypically low for gamma proteobacteria as *E. coli* strains carry 90–100 tRNA genes whereas *Pseudomonas aeruginosa* strains have roughly 55–64 tRNA genes. The mean number of rRNA clusters in gamma proteobacteria is 5.7. The plus strand rRNA locus of Sde2-40 has an unusual configuration with two divergently directed protein-encoding genes (Sde_1099 and Sde_1100) located between the 16S and 23S rRNA genes and the locus is flanked by apparent noncoding regions with uncharacteristically low G+C.

### Genome Evolution

The Sde 2-40 genome exhibits mosaicism in G+C content (Figure 1) suggestive of recently acquired segments from organisms with divergent genomes. It undergoes frequent cell/cell contact with other cells of the same species and even with eukaryotes. To identify horizontally acquired genes, protein-coding sequences for genes identified in the Sde 2-40 genome were evaluated using the CodonW algorithm for %G+C content, %G+C content of the third position of synonymous codons (GC3s) and for effective number of codons (Nc) as an indicator of codon usage. The %G+C content of protein-encoding genes was 46.3 ±3.6 with a disproportionate number of genes having low G+C. As the low %G+C genes tended to be clustered hypothetical genes of unassigned function, the reference gene set was defined as the subset of Sde 2-40 genes whose predicted function is basic metabolism. These also exhibited strong similarity to a homolog present in a pseudomonad (1057 comparable genes). This reference gene set exhibited a mean %G+C of 47.2±3.1, a GC3s of 43.8±5.8 and the Nc was 48.96±5.44. When these values were used to survey the genome, 35% of the genes (1412) were at least one standard deviation from the mean in at least two of the scored characters. Surprisingly only 23 out of 182 genes predicted to encode carbohydrases or CBM proteins exhibited this characteristic, suggesting that if recently acquired, the source organism had a genome similar to that of 2-40.

Several large clusters of divergent genes were identified in this survey that had characteristic features of integrons. The core integron was located between nt 526536 and 568028 and consisted of 40 genes expressed from the same strand. The integron was flanked by integron/phage integrases on each end. This region had a mean %G+C of 37.9±2.5 and all but three genes have unassigned functions. A 140 bp repeat was identified in each of the intergenic regions separating apparent transcriptional units that were >95% conserved. The same repeat was similarly located at five satellite sites in the genome (nt 2304665-2329512, 2953034-2974232, 3741500-3743893, 3937018-3943585, 4404545-4411107). These satellite regions also were low %G+C regions and most were on a flank, or internally in a transposase gene (exception: 3741500-3743893 region), suggesting that transposition of a repeat could generate a satellite integration site. Nearly all of the genes in these satellite clusters were also expressed from the same strand. There is some duplication of genes within these clusters but little synteny between them. Only Sde_0462 & Sde_0463 were syntenous with Sde_1818 & Sde_1819 suggesting that each of these clusters arose independently. For example, Sde_0426 is highly similar to Sde_0457 and Sde_1825. Sde_0456 and Sde_0458 are apparent duplicates of Sde_0427. Sde_0462 is homologous to Sde_1814, Sde_1818 and Sde_1830. Five other clusters of hypothetic genes were identified that lacked this repeat sequences (134591- 150065; 341150-354005; 418539-430371; 764673-773707; 3837720-3846061; 4133935-4137958) but were associated with an apparent transposase (DUF1568 homolog). In total, five potentially functional integron /phage integrases were identified in the genome that were associated with degenerate prophage (incomplete) or these integrons. Two integrase pseudogenes and three IS elements were also detected (ISSde_A, ISSde_B, ISSde_C).

### Megaproteins

The Sde 2-40 genome codes for 15 polypeptides longer than 2000 amino acids, ranging from 274 Kd to 1.6 Md. Each contains multiple domains and motifs that are reported to bind calcium and mediate protein/protein interactions [9],[10]. They are acidic, pI 3.5–4.9 and have a secretion signal. It is possible that proteins with these properties can function in binding prokaryotes to algae with associations between gamma proteobacteria and dinoflagellates reported [11]. These large proteins are unusual and help explain why the Sde 2-40 genome contains 5.06 Mb but codes for only 4008 genes, well below the general rule for prokaryotes of about one Kb/gene. The seven largest of these proteins are encoded by 120879 bases or approximately 2.4% of the genome.

### Signal Transduction

A common feature of each of the Sde 2-40 carbohydrase systems is their induced expression in response to their cognate substrate [8],[12],[13]. Many organisms with complex carbohydrase systems, constitutively express low levels of one or a few “sentinel” enzymes. The role of these sentinel enzymes is to release inducer molecules from the polysaccharide substrate which then activate one or more signal transduction systems to induce expression of the entire degradative pathway. This mechanism has been demonstrated experimentally for the cellulolytic fungus, *Trichoderma reesei* and the bacterium *Clostridium thermocellum* [14],[15],[16].

Each of the tested carbohydase systems exhibit classic glucose-dependent catabolite repression. *In silico* analysis confirms that the Sde 2-40 genome contains a strong homolog of adenylate cyclase (Sde_3600) and catabolite activator protein (Sde_0755) as well as the components of a phosphotransferase system (e.g. Sde_0348, Sde_3180, Sde_3182).

The genomic signal transduction profile of Sde 2-40 can be viewed in the MIST (Microbial Signal Transduction) database at http://genomics.ornl.gov/mist. Compared to other prokaryotic genomes, Sde 2-40 has approx. 40% more COG's devoted to signal transduction than other bacteria, (including the gamma proteobacteria; Table S1), although it has an average number of one- and two-component signal transduction regulatory systems for its genome size [17]. It also does not contain any unique sensor or regulator, however, some features of signal transduction do set the organism aside. These features are directly linked to its unique abilities to degrade complex polysaccharides. First, the genome is significantly enriched in regulators that control the level of cyclic di-guanylate, a second messenger, which determines the timing and amplitude of complex biological processes predominantly linked to the cell surface, such as exopolysaccharide biosynthesis and degradation and biofilm formation [18]. Cyclic di-GMP cyclases comprise the single largest signal transduction output domain family in the genome (more than 1% of the total genome content), although usually the most abundant output type in bacteria is one of the DNA-binding helix-turn-helix domains typical of transcription factors [17].

Another significant feature is the large proportion of membrane-bound one-component transcription factors. Less than 3% of bacterial one-component transcription factors are membrane-bound [17],[19], whereas in Sde 2-40 they comprise 20%. For example, 16 of the 31 AraC-type transcriptional regulators are membrane-bound in Sde 2-40, whereas all such regulators in a closely related species, *Shewanella oneidensis,* are soluble cytoplasmic proteins. AraC-type transcription factors frequently function as transcriptional activators of enzymes involved in catabolic pathways, although family members also activate or repress transcription of genes with a wide range of functions. The activity of AraC-like transcription factors is usually regulated allosterically by small molecules, such as the substrate for the first enzyme of a catabolic pathway.

Since bacterial transcriptional factors often directly regulate adjacent genes [20], we analyzed the genomic context in the vicinity of membrane-bound transcription factors and found several of them in the chromosomal proximity to diverse enzymes involved in cellulose degradation (Table 2). Most of these proteins are the result of the lineage-specific gene expansion; i.e. 13 of the 16 membrane-bound AraC-type transcriptional regulators are paralogs. We hypothesize that the unusual enrichment in membrane-bound transcription factors is an adaptive strategy for detecting extracellular complex polysaccharides and expressing genes necessary for its degradation.

**Table 2:** Membrane-bound one-component transcriptional factors encoded adjacent to genes for complex carbohydrate degradation.

Transcription factor (SDE locus^1^)	Number of TM regions^2^	Adjacent genes(SDE Locus^1^)
HTH_AraC (0324)	2	Cellulase (0325)
HTH_AraC (3613)	2	β-1,4-xylanase (3612)
HTH_AraC (2491)	6–7	Cellulase (2490)
HTH_AraC (2495)	6–7	1,4- β -glucosidase (2497)
HTH_AraC (2928)	6–7	Cellulase (2929)
HTH_AraC (3858)	6–7	Cellulose-binding protein; putative (3859)
HTH_LytTR (3422)	4	Cellulase (3420)

1 Gene number having the prefix “Sde”, for *Saccharophagus degradans* as assigned in Jun 15, 2005 genome assembly.

2 TM, abbreviation for predicted transmembrane regions.

The Sde 2-40 genome contains sets of genes for flagellar and type IV pili-based motility as well as regulatory systems for their control. There are two dedicated chemotaxis pathways predicted to control flagellar motility (anchored by chemotaxis histidine kinases CheA GI:90020168/Sde_0519 and GI:90021806/Sde_2161) and two chemotaxis-like pathways predicted to control type IV pili-based motility or other cellular functions (anchored by chemotaxis histidine kinases CheA GI:90022749/Sde_3107 and GI:90023269/Sde_3629). This implies that Sde 2-40 is not only capable of navigating to nutrient sources in water, but can also direct its motility across solid surfaces such as solid plant material. The chemotaxis signal transduction network contains 13 transducers for detecting both extracellular and intracellular signals. Interestingly, there are 8 predicted CheY response regulators. We hypothesize that diverse signals detected by chemotaxis transducers are distributed to control not only two types of motility apparatus, but also other cellular activities.

### Secretion Systems

The expression of the extraordinary array of secreted carbohydrases encoded by the Sde 2-40 genome requires the presence of robust protein secretion systems and, typical of proteobacteria, a complete Sec system was detected in its genome (Table S2). SignalP analysis revealed 1068 gene model products (26.6% of the gene models) that carry an apparent amino terminal signal sequence indicative of Sec-dependent secretion. The components for the Sec-associated SRP system for translocation of membrane proteins were also present. The Sde 2-40 SRP system appears to be unusual in that SRP54 M and G domains are located on separate polypeptides.

A twin arginine (Tat) secretion system was also identified. At least 15 gene products were annotated as being Tat secreted; most of these appear to be translocated to the periplasm. Components of both type I and type II secretion systems were also detected. Homologs of HlyB, HlyD and TolC, the essential components of a type I secretion system [21], were encoded by an apparent operon. Since type I secretion systems use a cryptic carboxy terminal secretion signal, proteins secreted by the type I system are not genomically obvious. However, there are several partial homologs of RTX toxins that are good candidates.

Three clusters of genes were identified that encoded components of the general secretory (Type II) pathway [22]. Cluster I consists of apparently co-transcribed gspCDEFGHIJKLMN that are organized similarly to the *Klebsiella* pul operon. A second cluster consists of gspD-G whereas the third was composed of gspEFH. A homolog to GspO, was also present that was independently transcribed. Although the Sde 2-40 genome carries two homologs of the *Klebsiella* PulA, neither is associated with a gsp cluster as observed in Klebsiella strains.

### The Degradative CAZome of *S. degradans*

The enzymatic breakdown of complex polysaccharides requires complex, multienzyme systems with diverse activities and substrate specificities. This complexity is required to overcome the chemical and structural complexities presented by complex polysaccharides and complex polysaccharide-containing structures (i.e. the plant cell wall). Among the fungi and bacteria, the vast majority of complex polysaccharide-degrading enzymes belong to different families of Glycosyl Hydrolases (GH). As classified by the Carbohydrate-Active Enzyme webserver (www.CAZY.org/index.html), GHs are assigned to 112 sequence-based families. A distinctive feature of the GHs that attack complex insoluble polysaccharides is their modular architecture, in which the catalytic module(s) is linked to one or more non-catalytic modules.

The most common type of non-catalytic components are high-affinity substrate binding modules (carbohydrate-binding modules; CBMs) which act to bring the enzymes into intimate and prolonged association with their complex substrates. They potentiate catalysis by reducing the access limitation imposed by the composite structure of the complex polysaccharide. These CBMs, grouped into approximately 50 sequence based families, specifically target the enzyme to its proper substrate amongst the chemical and structural complexities of the plant cell wall. Furthermore, CBMs allow soluble enzymes to remain in contact with their insoluble substrates in an aqueous environment [23].

**Figure 2:**
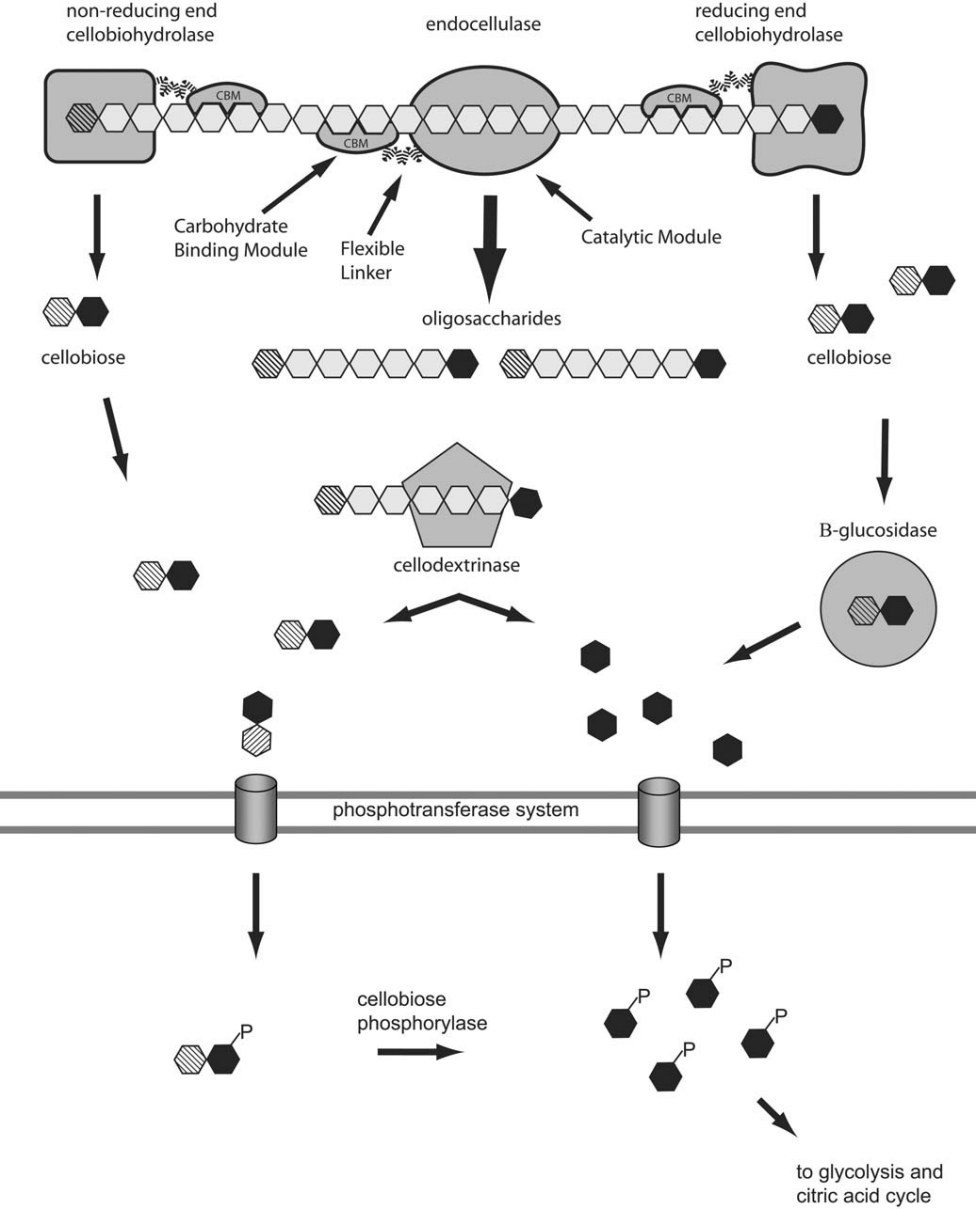
Schematic organization of a typical Glycosyl Hydrolase catalyzing endo-cleavage of a polysaccharide shown within a generic cellulase system pathway. Carbohydrate-binding modules (CBMs) specifically target enzymes to their substrates, initiating and maintaining prolonged contact with the insoluble polysaccharide. The catalytic module may be a glycosyl hydrolase (GH) polysachharide lysase (PL), glycosyl transferase or an esterase. The flexible linker affords the catalytic module a certain freedom of movement, which presumably allows it to adjust to conformational variations in the substrate while the CBM maintains contact with the substrate. Enzymes, representative of a typical cellulase system, are depicted depolymerizing a single cellulose chain. Exo-acting cellobiohydrolases and endoglucanase synergistically degrade polymeric cellulose to cellobiose and cellodextrins, respectively. At least part of the synergism is believed to result from the activity of endoglucanases creating additional ends for exoglucanases to act upon. Cellodextrins (soluble cello-oligomers) may be further processed to glucose and cellobiose by cellodextrinases. Depending on the organism cellobiose may be cleaved extracellularly by β-glucosidases (cellobiases) and imported as glucose, or imported directly and cleaved in the cytoplasm. Import generally occurs through phosphotransferase transport systems, resulting in cytoplasmic Glucose-6-Phosphate (G6P) and phosphorylated cellobiose. Certain organisms, such as *Clostridium thermocellum*, are also capable of importing cellodextrins for cytoplasmic cleavage. Systems that degrade other complex polysaccharides (e.g. chitin) share many of the features depicted for cellulose degradation, i.e. endo- and exo-acting enzymes and polymer-specific CBMs; however, there are substrate-specific variations in enzymatic composition, to include enzymes dedicated to the removal of side-chains such as xylose and/or arabinose oligomers or substituent groups, which may include acetate, sulfate and methyl, among others.

**Figure 3:**
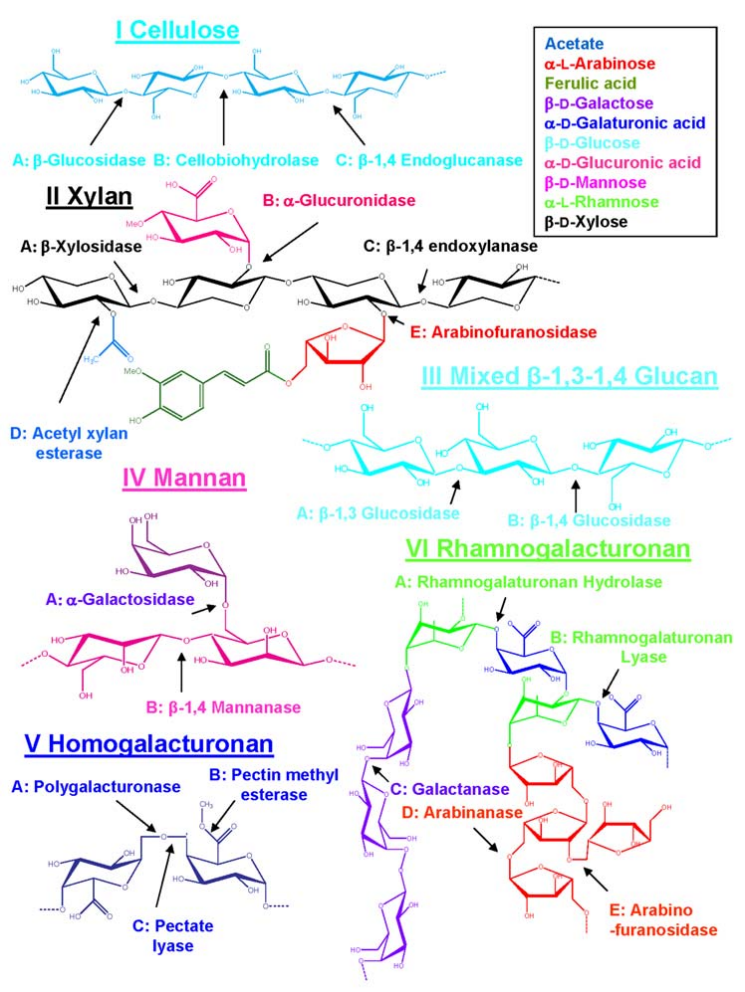
Oligimers of six major types of complex polysaccharides (Roman numerals), their component sugars and enzymes (Capital letters) that act on specific bonds within the molecules. The figure is keyed to Table 5, Enzyme activity induced by sole carbon complex polysaccharides. Sugars are color coded and keyed in box.

Polysaccharide degrading systems often contain multiple enzymes with synergistic activities. For example, most characterized cellulase systems contain multiple endo-acting enzymes and one or more exo-acting cellulases, which usually liberate cellobiose from cellulose chains (cellobiohydrolases). The activity of the endoglucanases provides more free chain ends to be acted on by the cellobiohydrolases. Most systems also incorporate “accessory enzymes” such as cellodextrinases and cellobiases to achieve complete breakdown to usable monomers [24],[25]. Figure 2 illustrates the modular components of a typical GH and presents a generic cellulose degradation pathway. Specific substrates and carbohydrases are shown in Figure 3.

With genes encoding 128 glycoside hydrolases (GHs), Sde 2-40 is one of the most prolific bacteria sequenced; to date it ranks 3^rd^ of almost 400 bacterial genomes surveyed by the Carbohydrate-active enzymes database (www.cazy.org/CAZY). Most carbohydrase-coding ORF's are dispersed throughout the genome, however, some are clustered, including amylases (Sde_0556-Sde_0601), arabinoxylosidases (Sde_0777-Sde_0789), pectinases (Sde_0937-Sde_0953), and alginases (Sde_3272-Sde_3286).

The set of glycosidases encoded by Sde 2-40 is particularly adapted to the degradation of a multitude of plant and algal cell wall polysaccharides and is characterized by an extreme modularity in the structure of the enzymes (see Figure 2; Figures S1, S2, and S3). Substrates catabolized include cellulose, substituted xylans, xyloglucans, arabinans and arabinogalactans; pectin and rhamnogalcturonan; β-1,3(4) glucan, β-1,3-glucans; starch, glycogen, pullulan; mannans, glucomannans and galactomannans. Interestingly, the xylan, mannan and cellulose degrading apparatus of Sde 2-40 is very similar to the reported cellulases, xylanases and mannanases synthesized by a related terrestrial gram negative bacterium, *Cellvibrio japonicus* (for review see [26]). The similarity between the GH and CBM (see below) composition of these enzyme systems in Sde 2-40 and *C. japonicus* indicate a close evolutionary relationship between the plant cell wall degrading apparatus in the two organisms. The etiology of such a relationship remains a compelling area for study.

In addition to the cellulose, xylan and mannan degrading enzymes described above, the Sde 2-40 genome also encodes eight endo-β-1,3-glucanases (Table 3), each of which has a type II secretion signal sequence, interesting domain architecture and modules [viz. thrombospondin type 3 (TSP3) and cadherin-like (CADG) calcium-binding motifs]. It is also noteworthy that the Sde 2-40 genome codes for 33 polysaccharide lyases (PLs; Figure S4), far more than any other bacterium including *B. thetaiotamicron* (15), *Erwinia carotovora* (13) and *Pseudomonas syringae pv. phaseolicola* (8) and any fungus. The only organisms that have (marginally) more PLs than Sde 2-40 are the plants (*Arabidopsis* has 34 and the poplar has 39) whose higher number is entirely attributable to large multigene families. Twenty three of the 33 Sde 2-40 PLs are modular (a proportion never seen before). Significant domain structures are shown in Figure S4.

**Table 3:** Genomically predicted laminarinases.

Name	Locus Tag^1^	Predicted function^2^	Modules^2^,^3^	amino acids^4^	MW^4^
Lam16A	1393	β-1,3-glucanase	GH16/CBM6/CBM6/TSP3/TSP3/TSP3/TSP3/COG3488	1,707	163.3
Lam16B	2927	β-1,3-glucanase	GH16/CBM6/CBM6/EPR(56)/CBM32/CBM32	1,441	158.6
Lam16C	1444	β-1,3-glucanase	GH16/CBM4/CBM32/CBM32	1,184	129.1
Lam16D	3021	β-1,3-glucanase	GH16/CBM32/PSL(48)/TMR	722	77.7
Lam16E	0652	β-1,3-glucanase	CBM6/CBM6/GH16	569	61.4
Lam16F	3121	β-1,3-glucanase	LPB/GH16	742	80.2
Lam16G	2832	catalytic residues missing	LPB/GH16/CBM6/CBM6	877	94.2
Lam81A	2834	β-1,3-glucanase	LPB/CAD/GH81/FN3/FN3	1,238	133.1

1 Gene number having the prefix “Sde”, for *Saccharophagus degradans* as assigned in Jun 15, 2005 genome assembly. (http://genome.ornl.gov/microbial/mdeg/15jun05/mdeg.html).

2 Predictions of function and module determination by CAZy ModO at AFMB-CNRS.

3 Module abbreviations: CAD, cadherin-like domain; CBM, carbohydrate binding module; COG3488, thiol-oxidoreductase like domain; EPR, glutamic acid-proline rich region; FN3, fibronectin type 3 module; GH, glycoside hydrolase; LPB, lipobox signature sequence; PSL, polyserine linker; TMR, transmembrane region; TSP3, thrombospondin type 3 repeat.

4 MW and amino acid count calculated using the protParam tool at http://us.expasy.org/tools/ based on DOE/JGI gene model amino acid sequence translations including any predicted signal peptide.

Forty three of the 128 GHs encoded by the genome are appended to at least one known CBM. More than one-quarter of the >180 carbohydrate depolymerases have polyserine repeat regions (PSL) separating functional domains [27], rather than the proline/threonine sequences that link the modules of GHs in other prokaryotes. PSL are also evident in the plant cell wall degrading enzymes of *C. japonicus* [26],[28], again pointing to a close evolutionary relationship between the plant cell wall degrading apparatus of the two bacteria.

It is possible that the large number of CBM-containing enzymes reflects the chemical complexity of the marine plant cell wall as compared to the corresponding terrestrial structures. However, it seems more likely that the apparent requirement for secreted enzymes to contain CBMs in the marine bacterium, may reflect the aqueous nature of the environment. Thus, in such a dilute ecosystem, if secreted enzymes are not tethered to the plant cell wall via CBMs they will rapidly disperse and their benefit to the host organism will be lost. Indeed, the critical “tethering” role of the CBMs may select for modules with high affinity for the plant cell wall. In GHs that contain multiple CBMs very tight affinity for targeted polysaccharides can be achieved through avidity effects between the modules [29],[30]. Indeed, the evolutionary driver for the multiple CBMs in the extracellular Sde 2-40 enzymes could be the requirement for these GHs to be in continued contact with the plant cells preventing their dissipation into the aqueous marine environment. It is also possible that the presence of multiple CBMs is the result of adaptation to the high-ionic strength environment presented in marine and estuarine environments.

The genome of Sde 2-40 encodes the largest set of identifiable CBMs (127) reported in any organism sequenced so far. It has more than *Arabidopsis thaliana* (92) the fungus *Magnaporthe grisea* (66), *Homo sapiens* (35) and the huge poplar genome (116); and far more than any other bacterial species.

A distinctive feature of the Sde 2-40 GHs is the prevalence of enzymes that contain both a CBM2 and CBM10, modules that bind to crystalline cellulose. Indeed, as cellulose is the most abundant polysaccharide in plant cell walls, this may explain why the cognate degradative enzymes exploit this polymer as a universal receptor. It is interesting to note that a large number of the *C. japonicus* plant cell wall-degrading GHs, reported to date, also contain CBM2 and/or CBM10 modules, again suggesting a close evolutionary relationship between the marine and terrestrial bacterium that was not anticipated.

In addition to CBM2s and CBM10s, there is a dramatic expansion of modules in CBM families 6 and 32 in Sde 2-40, which contains 39 and 25 members, respectively. It is likely that the marine environment has imposed selective pressure that has led to the expansion of these two CBM families as several lyases that attack the marine polysaccharide alginate contain CBM32s, while two GH16 agarases contains several CBM6s. Indeed, both CBM families 6 and 32 have been shown to display flexible ligand specificity with the former family recognizing polymers containing D-glucose, D-xylose, D-galactose and 3,6 dehydro-L-galactose [31],[32],[33], while members of the latter family interact with galactose-containing carbohydrates that are modified at C2 (N-acetylgalactosamine) or C6 (galacturonic acid) [34]. As marine polysaccharides contain a wider range of sugars (for example alginate contains D-mannonic acid and L-guluronic acid and agarose contains 3,6 dehydro-L-galactose) than terrestrial plant cell walls, the expansion of family 6 and family 32 CBMs is consistent with the diversity of sugar polymers encountered by Sde 2-40. As *C. japonicus* does not occupy an environmental niche that contains marine polysaccharides it is unlikely to contain similarly large numbers of family 6 and family CBMs.

Additionally the Sde 2-40 genome codes for many novel combinations of CBMs and catalytic domains, observed for the first time (see Figures S1, S2, S3, and S4). Many other proteins have modules of unknown function appended to the catalytic domain. Several proteins have two catalytic domains: Sde_3061; Sde_3870; Sde_3003; Sde_3612; Sde_0943; Sde_2873. In Figures S1, S2, S3 and S4 the boxed proteins have CBMs attached to domains of completely unknown function illustrating (i) the benefit of whole genome sequencing (without which it would have been difficult to identify these proteins) and (ii) that our knowledge of the plant cell wall degradome is far from complete. These proteins will therefore constitute targets of choice for subsequent functional studies.

### Functional Genomics with a Focus on Carbohydrase Regulation

Functional characterizations of the agarase [12], chitinase [35],[36], cellulase [13] and alginase (11 enzymes; [37]) systems of Sde 2-40 showed that each degraded the respective complex polysaccharides to monomers. It was discovered that five agarases were distributed among three GH families (GH16, GH50, GH86; [12]) and that two of the agar depolymerases contained novel CBM6 modules with interesting affinities [33].

In order to evaluate the expression of each major carbohydrase system during growth on specific carbon sources, transcript levels for genes encoding a selected carbohydrase from each system were estimated by qRT-PCR (Table 4). Each monitored gene exhibited low basal expression during growth on glucose supporting the notion of an operational global catabolite repression mechanism. Transition to another carbon source resulted in a slight increase in expression consistent with release of glucose-dependent catabolite repression. The highest expression, however, was observed on the substrate associated with each carbohydrase. For example, transcript levels for the agarase Aga16B increased 280-fold after 4 hr growth on 0.1% agar. This infers that the expression of each these systems is regulated by signal molecules released from the corresponding substate. Some apparent cross talk between regulatory systems was observed, particularly with those complex polysaccharides that are interlaced with other complex polysaccharides in nature. Thus, growth on microcrystalline cellulose (Avicel) also induced expression of the monitored xylanase, *xyn11A*. Similarly growth on xylan induced *cel5H* expression.

**Table 4:** Substrate-specific induction of selected carbohydrase genes.

Gene^1^	Relative Transcript Levels after Growth on^2^:					
	Agar	Alginate	Avicel	Chitin	Xylan	Glucose
*aga16B*	284±46	4±1	2±0.5	22±3	88±22	0.5±0.1
*algF*	1±0.5	31±1	0.5±0.1	2±0.5	10±0.5	1±0.1
*cel5H*	6±2	3±0.5	778±200	201±57	158±50	0.5±0.1
*chi18A*	1±0.5	4±1	3±0.5	1749±146	102±20	2±0.5
*xyn11A*	9±2	3±1	292±50	1057±10	1349±191	0.5±0.1

1 *aga16B*, Sde_1175; *algF*, Sde_2873; *cel5H*, Sde_3237; *chi18A*, Sde_1704; *xyn11A*, Sde_0701

2 *S. degradans* 2-40 was grown in minimal medium supplemented by 0.2% glucose to an OD_600_ of 0.33–0.35. The cells were harvested and transferred into fresh medium containing 0.2% xylan, chitin, alginic acid, Avicel, or glucose or 0.1% agar as indicated. After 4 hr, total RNA was extracted, converted to a cDNA copy and transcript levels relative to initial levels estimated by qRT-PCR using gene-specific primer pairs as described in the Materials and Methods. Transcript levels were normalized to guanylate kinase (Sde_3695) transcript levels.

We also examined enzyme activity in response to sole carbon complex polysaccharide inducers. As in the case of the qRT-PCR studies, we predicted that substrates that signal the presence of complex material, such as the plant cell wall, would trigger a general, extensive enzyme response. In cases where the complex polysaccharide does not occur as a part of a multi- complex polysaccharide complex, it was predicted that enzyme induction would be more specific. As anticipated, growth on *Spartina alterniflora* leaves induced enzyme activities against all tested substrates (Table 5; Figure 3): crystalline cellulose (Avicel), amorphous cellulose (PASC and CMC), xylan, β-1,3,4-glucan (barley β-glucan) and β-1,3-glucan (laminarin). Interestingly, growth on Avicel and xylan also broadly induced enzymes. These results indicate that cellulose and xylan could function as plant cell wall specific signature molecules, inducing a full suite of degradative enzymes required for deconstruction of plant material. This stands in contrast to the more specific patterns of induction when Sde 2-40 was grown on barley glucan or laminarin. Neither substrate induced activity against xylan or Avicel. The presence of low, but detectable, levels of activity against CMC and laminarin in glucose-grown cultures suggests that the cellulase system of Sde 2-40 utilizes sentinel enzymes, and that these enzymes have activity against β-1,4- and β-1,3-glucans.

**Table 5:** Enzyme activity induced by sole carbon complex polysaccharides (CP).

Growth medium sole Carbon Source substrate (inducer)						
Activity vs.	*Spartina* 1 leaves	Avicel^2^	Xylan^3^	β-glucan^4^	Laminarin^5^	Glucose
CP (see Figure 3)	I-VI	I	II	III	III	N/A
Avicel^2^	4.8	10.5	93	0	0	0
CMC^6^	58.2	218	117.9	95.5	59.6	7.2
PASC^7^	51.1	254.2	118.3	0	37.5	0
Xylan^3^	29.8	111.1	267.2	0	0	0
β-glucan^4^	33.7	157.6	169	203.2	159.3	0
Laminarin^5^	28.8	72.5	101.4	164.3	295.1	50.2
Carbohydrases required to degrade substrate(keyed to Figure 3)	cellulases (I:ABC) xylanases (II:ABCDE) β-glucanases (III:AB) mannanases (IV: AB) pectinases (V:ABC VI:ABCDE)	Cellulases (I:ABC)	xylanases (II:ABCDE)	β-1,3 and β-1,4-glucosidases (III:AB)	β-1,3-glucosidases (IIIA)	Glycolysis, TCA cycle

1 *Spartina alterniflora* (saltmarsh cord grass), found in intertidal wetlands, has a cell wall with approx. 10% lignin, with the remainder being hemicellulose, cellulose, and pectin

2 Purified cellulose, ∼70% crystallinity

3 Birchwood xylan, glucuronoarabinoxylan

4 Barley β-glucan, mixed β-1,3- and β-1,4-glucan

5 *Laminaria digitata* laminarin, β-1,3-glucan

6 Carboxymethyl cellulose, 100% amorphous

7 Phosphoric acid swollen cellulose, intermediate crystallinity between Avicel and CMC

We analyzed spent growth media by mass spectrometry (MS) analysis to identify carbohydrases expressed and exported by Sde 2-40 cultivated in avicel or xylan as sole carbon sources. The MS analysis supported the enzyme induction studies. For example, many cellulases and xylanases were induced by their homologous substrate; others, e.g. cellodextrinase Ced3A were induced by several different substrates (Table 6) [38]. From the genomic, qRT-PCR, enzyme activity and MS studies, we conclude that: 1- the Sde 2-40 carbohydrases are regulated by multiple mechanisms; 2- the more complicated the polysaccharide complex, the more enzyme systems are induced; 3- glucose repression is a key regulation mechanism.

**Table 6:** Examples of carbohydrases and CBM proteins detected in *S. degradans* supernatants by mass spectrometry.

Growth Substrate^1^	Name	Predicted function^2^	Modules^3^	Locus Tag^4^	amino acids^5^	MW^5^
Avicel	Cel5H	endocellulase	GH5/PSL(32)/CBM6/EPR(16)	3237	630	66.9
	Cel5I	endocellulase	CBM2/PSL(33)/CBM10/PSL(58)/GH5	3420	725	77.2
	Cel9B	endocellulase	GH9/PSL(54)/CBM10/PSL(50)/CBM2	0649	867	89.5
	Ced3A	cellodextrinase	LPB/GH3	2497	1,072	116.0
	Xyl3A	β-xylosidase	LPB/GH3	1487	893	97.6
	Cbm2B	cbm only	CBM2/UNK(914)	1183	1,042	112.1
Xylan	Xyn10E	β-xylanase	LPB/EPR(47)/GH10	0323	670	75.2
	Xyl3A	β-xylosidase	LPB/GH3	1487	893	97.6
	Xyl31A	α-xylosidase	LPB/GH31	2500	973	110.2
	Ced3A	cellodextrinase	LPB/GH3	2497	1,072	116.0
	Ced3B	cellodextrinase	LPB/GH3	0245	862	92.9
	Cep94B	cellodextrin phosphorylase	GH94	0906	788	88.7
	Gly3D	β-glycosidase	CBM32/CBM32/CBM32/GH3/CBM32	0475	1,581	173.0
	Cbm2C	cbm only	CBM2/PSL(58)/Y94/PSL(25)/UNK(577)	0182	933	97.5
	Cbm32A	cbm only	CBM32/CBM32/UNK(251)	0478	1,028	111.9

1 Protein was detected in supernatants of cultures grown in the following growth substrates: Avicel (∼70% crystalline cellulose), xylan (Birchwood glucuronoarabinoxylan).

2 Predictions of function and module determination utilizing the routines used for the updates of the CAZy database (www.cazy.org/CAZY/).

3 Module abbreviations: CBM, carbohydrate binding module; UNK, unknown function; PSL, polyserine linker; LPB, lipobox signature sequence; GH, glycosyl hydrolase; EPR, glutamate-proline rich region.

4 Gene number having the prefix “Sde”, for *Saccharophagus degradans* as assigned in Jun 15, 2005 genome assembly

5 MW and amino acid count calculated using the protParam tool at http://us.expasy.org/tools/ based on DOE/JGI gene model amino acid sequence translations including any predicted signal peptide.

The carbohydrases were shown to be functional in microcosms as Sde 2-40 grew on plants as sole carbon sources while fully degrading them, being the first marine prokaryote shown to do so [38]. One line of evidence came from growth studies showing that Sde 2-40 did not grow in minimal medium (MM) lacking a carbon source whereas it underwent numerous generations (g) in MM+0.2% glucose (51 min gt), MM+washed, dried, sterile, *Spartina alterniflora* leaves (280 min gt), Avicel, xylan or filter paper. Growth was concurrent with pronounced degradation of the plant/CP.

Notably, Sde 2-40 degraded a variety of cellulositic plants in monoculture rather than as part of a consortium, having ORFs that annotate as putative ligninases including a polyphenol oxidase Sde0315, a tyrosinase Sde0316, and three peroxidases Sde0090, Sde2430, and Sde2810. There was also physiological evidence of Sde 2-40 ligninase activity; for example, it degraded Remazol Brilliant Blue R (RBBR) and poly B 411, two indicator dyes for fungal ligninase activity.

### Cell Biology

It is becoming increasingly clear that spatial placement significantly impacts enzyme function. Gram-positive clostridia make cellulosomes, multi-enzyme complexes, mediated by intermodular dockerin/cohesin interactions, which bring to bear an organized array of cellulases to exocellularly depolymerize substrates, conserving both enzyme and substrate [39],[40]. Sde 2-40 utilizes at least one Gram-negative solution to the same problem, i.e. post-translationally modified lipobox domains that anchor proteins to the outer membrane. The database of bacterial lipoproteins (DOLOP) analysis revealed that 34 genes contain lipobox sequences, 31 of which are predicted carbohydrases.

While lipoprotein-anchors of have been well studied [41],[42],[43] and reported to be a strategy for surface attachment of degradative enzymes [39], the mechanism had only been reported to pertain to only a few proteins per cell. This report is the initial finding of extensive involvement of the motif, encompassing at least one degradase per carbohydrase system including, cellulases (5) [13], pectinases (5), xylanases (5), chitinase(1) [36], agarase (1) [12], laminarinase (1), and mannanase (1). The 34 predicted carbohydrases or CBM proteins which carry lipobox sequences amount to 15% of the total ORFs predicted to degrade or bind carbohydrates in Sde 2-40.

Furthermore, carbohydrases are believed to be anchored to the outer membrane by more than one mechanism. The cell surface of Sde 2-40 is smooth in its logarithmic phase of growth when growing on glucose [3] and becomes nodulated when growing in CP or are starving (Figure S5). These protuberances could be indicative of protein/protein interactions that anchor certain enzymes at the cell surface. It is interesting to note that two Sde 2-40 ORFs appear to contain dockerin-like motifs and six others contain one or two putative, but distantly related, cohesin-like module(s). In future studies, it will be of interest to explore whether recombinant forms of the dockerins bind to any of the candidate cohesin-like modules, considering Sde 2-40 does not contain a classical cellulosome system.

### Conclusions

Consortia of microorganisms are usually required to degrade complex carbohydrates, e.g. cellulose, and such microorganisms are usually specialists in the degradation of one or a few different carbohydrates. Sde 2-40 is unique in its array of different carbohydrases, and unusual in its ability to *completely* mineralize a plant, in pure culture, in marine waters. Given the additional abilities of Sde 2-40 to degrade algal structural polymers (agar and alginate) as well as the invertebrate polysaccharide, chitin, the bacterium may well have an important role in the marine carbon cycle.

## Materials and Methods

### Sequencing Strategy

Whole genome shotgun sequencing and finishing were carried out by the US Department of Energy Joint Genome Institute (JGI). All complete library construction and sequencing protocols can be found at: http://www.jgi.doe.gov/sequencing/protocols/index.html. Briefly, genomic DNA was randomly sheared with a hydroshear device (Genemachines, San Carlos, CA) and fragments were blunt-end repaired using T4 polymerase and Klenow fragment. Fragments were size selected by agarose gel electrophoresis and ligated into pUC18 (∼3 kb inserts), pMCL200 (∼7 kb inserts) or ∼35 kb inserts in pCC1Fos (Epicentre, Madison, WI). Ligations were transformed into *E.coli* DH10B cells and colonies were picked into 384-well plates containing LB and glycerol. DNA for sequencing was produced by rolling circle amplification (Templiphi, GE Healthcare, Piscataway, NJ) or Sprintprep (Agencourt, Beverly MA) magnetic bead DNA purification. Subclone inserts were sequenced from both ends using universal primers and ET (GE Healthsciences, Piscataway, NJ) or Big Dye (ABI, Foster City, CA) terminator chemistry. Approximately 144,000 sequence reads were assembled with the Phred/Phrad/Consed software package [44] resulting in approximately 16X coverage of the assembled genome. Finishing was performed by resolving repeats and gap closure using PCR, custom primer reactions, and manual editing. The resulting finished sequence is calculated to contain less than 1 in 50,000 errors with no gaps in the sequence.

### Genome Annotation

Automated gene prediction was performed using the output of Critica [45] complemented with the output of Generation and Glimmer [46], and is available at http://genome.ornl.gov/microbial/mdeg/. The tRNAScanSE tool [47] was used to find tRNA genes, while ribosomal RNAs were found using BLASTn vs. the 16S and 23S ribosomal RNA databases. Other “standard” structural RNAs (e.g., 5S rRNA, rnpB, tmRNA, SRP RNA) were found using covariance models with the Infernal search tool [48]. The automatic assignment of product descriptions was made using search results of the following curated databases in this order: TIGRFam; PRIAM (e^−30^ cutoff); Pfam; Smart; COGs (e^−10^ cutoff); Swissprot/TrEMBL (SPTR); KEGG. If there was no significant similarity to any protein in another organism, it was described as “Hypothetical protein”. “Conserved hypothetical protein” designated at least one match to a hypothetical protein in another organism. EC numbering was based on searches in PRIAM at an e^−10^ cutoff; COG and KEGG functional classifications were based on homology searches in the respective databases. Some enzymes were manually curated. In particular, all carbohydrate-degrading enzymes were detected and annotated by comparison to the Carbohydrate-active enzymes database (http://www.cazy.org/CAZY).

### Data Analysis

Phylogenetic and molecular evolutionary analyses were conducted using MEGA version 3.1 [49]. The percent G+C content (%G+C) of gene models, %G+C at the third position of synonymous codons (GC3s), and the effective number of codons (Nc) for all 4008 candidate protein-encoding gene models were determined using CodonW 1.4.2 (http://codonw.sourceforge.net/). CodonW output, along with information from the ORNL annotation files, were compiled and imported into Microsoft Excel 2000. Truth tables were constructed. For each sequence characteristic, the candidate protein-encoding gene's distance values from the mean were converted to Boolean values based on the presence of the distance value within a predefined range (e.g. greater than or equal to two standard deviations higher than the mean). The candidate protein-encoding gene models' best hits against the GenBank NR protein database were converted to Boolean variables based on whether or not the closest match was *Hahella chejuensis* KCTC 2396, and another variable was similarly assigned to reflect if the closest match was to a sequence in the *Pseudomonas* genus. These variables were used to count and sort candidate protein-encoding gene's based on NR database sequence similarity and/or distance from the mean for a particular sequence characteristic.

Integron repeat locations were assessed with Artemis Comparison Tool [50] comparing the 2-40 genome against itself with the percent identity cutoff reduced to 99% to eliminate self matches. Areas containing repeats were extracted and imported into ClustalX 1.83 [51] for alignment. Alignments were manually inspected and trimmed to include only sequence conserved across all aligned sequences. A hidden Markov model [52] was constructed using HMMbuild and a consensus sequence was determined through HMMemit (both programs from the HMMER package available from http://hmmer.wustl.edu/). The sequences were compared to the consensus sequence, and a representative sequence was chosen based on similarity to the consensus sequence. The Stand-alone BLAST package (binaries available from ftp://ftp.ncbi.nih.gov/blast/executables/) was used to identify repeat locations and integron cassette homologs. The formatdb program from this package was used to build a searchable database from the Sde 2-40 genomic sequence, while the blastall program was used to search this database using the query. Homologous cassettes were found to be end-to-end matches (including flanking repeats) with approximately 90% identity or greater.

### Bacterial Growth Media and Conditions

*E. coli* strains were grown under standard protocols. Sde 2-40 strain 2-40 (ATCC43961T) was grown at 27° C on half strength Marine Agar (MA): 18.7 g/L Difco Marine Broth 2216 amended with 1.5% agar or in minimal broth medium (MM) consisting of (per L): 23 g Instant Ocean Sea salts (Aquarium systems, Mentor, OH ), 1 g Yeast extract, 50 mM Tris buffer pH 7.4 and 0.05% (w/v) NH4Cl. MM was supplemented with 0.2% (w/v) Avicel, barley glucan, laminarin, or xylan.

### qRT-PCR

RNAprotect Bacteria Reagent was mixed with an aliquot of the cell culture at a 2∶1 ratio. After incubation at room temperature for 5 min, the cell suspension was harvested and RNA purified by using the Rneasy Mini kit (Qiagen). A cDNA copy was generated using the QiantiTect Reverse Transcription Kit according to the manufacturer's instruction. Primer pairs were designed to amplify 120 and 180 bp regions internal to the open reading frame of the gene being investigated. The housekeeping gene guanylate kinase (GK) was selected as control. The 20 µl qualitative RT-PCR reaction system contains 10 µl of 2× LightCycler 480 SYBR Green Master, 1 µl of cDNA, 1 µl of each 5 µM forward and reverse primers and 7 µl of dH_2_O. Real-time PCR was performed on a Light Cycler 480 (Roche), according to the manufacturer's instruction. Cycling conditions were as follows: initial denaturation at 95°C for 4 min and 45 cycles of 95°C for 15 s, 56°C for 15 s and 72°C for 20 s.

### Enzyme Induction and Activity Studies

Sde 2-40 was grown in 1 L shake flasks containing MM amended to 0.2% (wt/vol) dried *Spartina alterniflora* leaves, Avicel, birchwood xylan, barley beta-glucan, laminarin, or glucose. *Spartina alterniflora* (saltmarsh cord grass), found in intertidal wetlands, has a cell wall with approx. 10% lignin, with the remainder being hemicellulose, cellulose, and pectin. Cultures were grown to stationary phase and harvested by centrifugation. Estimates of cellular and supernatant protein were performed using the Pierce BCA assay. Cell and supernatant fractions were analyzed by the microplate adaptation of the Nelson-Somogyi reducing-sugar assay [53]. Samples were assayed for activity against Avicel, PASC, CMC, xylan, beta-glucan and laminarin. Activities were calculated as U/mg protein, where 1 U = 1 µmol reducing sugar equivalent released/minute. Activities reported in this study represent the sum of cell pellet and supernatant activities in U/ml.

### Mass Spectrometry and Proteomic Analyses

Supernatants of Avicel, CMC, and xylan- grown cultures were concentrated to ∼25X in Centricon or Microcon devices (Millipore). Protein concentrations were determined by the BCA protein assay (Pierce). Samples were denatured and reduced, alkylated in 50 mM iodoacetate and digested overnight at 37°C with proteomics grade trypsin (Promega). Digestions were analyzed by RPHPLC-MS/MS at the UMCP College of Life Sciences CORE Mass Spectrometry facility as previously described [13]. All peptide fragment masses were analyzed by the peptide analysis packages SEQUEST and MASCOT [54],[55] and compared to amino acid sequence translations of all gene models in the Sde 2-40 draft genome and to the non-redundant Mass Spectrometry Database (ftp://ftp.ncbi.nih.gov/repository/MSDB/msdb.nam).

### Supporting Information

All supporting information (Table S1 and S2; Figures S1, S2, S3, S4, and S5) is available on the PLoS web site, www.plosgenetics.org. The automated annotation and supporting information are available on http://genome.jgi-psf.org/mic_home.html.

## Supporting Information

Figure S1 *S. degradans* proteins carrying CBM2 domains. Asterisks identify novel combinations of CBMs and catalytic domains. The boxed proteins have CBMs attached to domains of, as yet, completely unknown function.
(0.07 MB PPT)

Figure S2 *S. degradans* proteins carrying CBM6 domains. Asterisks identify novel combinations of CBMs and catalytic domains. The boxed proteins have CBMs attached to domains of, as yet, completely unknown function.
(0.07 MB PPT)

Figure S3 *S. degradans* proteins carrying CBM13 domains. Asterisks identify novel combinations of CBMs and catalytic domains.
(0.06 MB PPT)

Figure S4 *S. degradans* modular polysaccharide lyases. 23 of the 33 Sde 2-40 PLs are modular, a higher proportion than observed in any organism thus far. Asterisks identify novel combinations of CBMs and catalytic domains.
(0.06 MB PPT)

Figure S5 Scanning electron micrographs of *S. degradans* grown in minimal agarose medium. Cells were harvested at the indicated growth stage, washed twice and resuspended in 20 mM PIPES buffer, pH 6.8, amended to 1% final concentration glutaraldehyde and immobilized onto. 0.2 µm pore size Nucleopore 13 mm polycarbonate filters (Whatman, Middlesex, UK) followed by post-fixing in 2% (v/v) osmium tetraoxide (OsO4) and dehydration in a standard ethanol series. After critical point drying in CO2, the specimens were mounted and coated with ∼10 nm gold/palladium. Specimens were viewed on a Hitachi S-4700 ultra high resolution scanning electron microscope (UHR-SEM). A) Cell of S. degradans grown to mid-log phase exhibiting typical morphology and surface topology consisting of knobs at the polar termini and large, irregular surface protuberances. B) Late-stationary phase cell having typical shortened morphology and abundant smaller protuberances and apparent fibrilar appendages.
(1.02 MB PPT)

Table S1 Frequency of COG's in *S. degradans* 2-40.
(0.05 MB DOC)

Table S2 Homologs of protein secretion system components.
(0.07 MB DOC)
