# Supplementary figures and images for: Complete Genome Sequence of the Complex Carbohydrate-Degrading Marine Bacterium, Saccharophagus degradans Strain 2-40T
Source: PLoS Genet. 2008 May 30;4(5):e1000087. doi: 10.1371/journal.pgen.1000087 (PMC2386152; doi:10.1371/journal.pgen.1000087)

## Slide 1
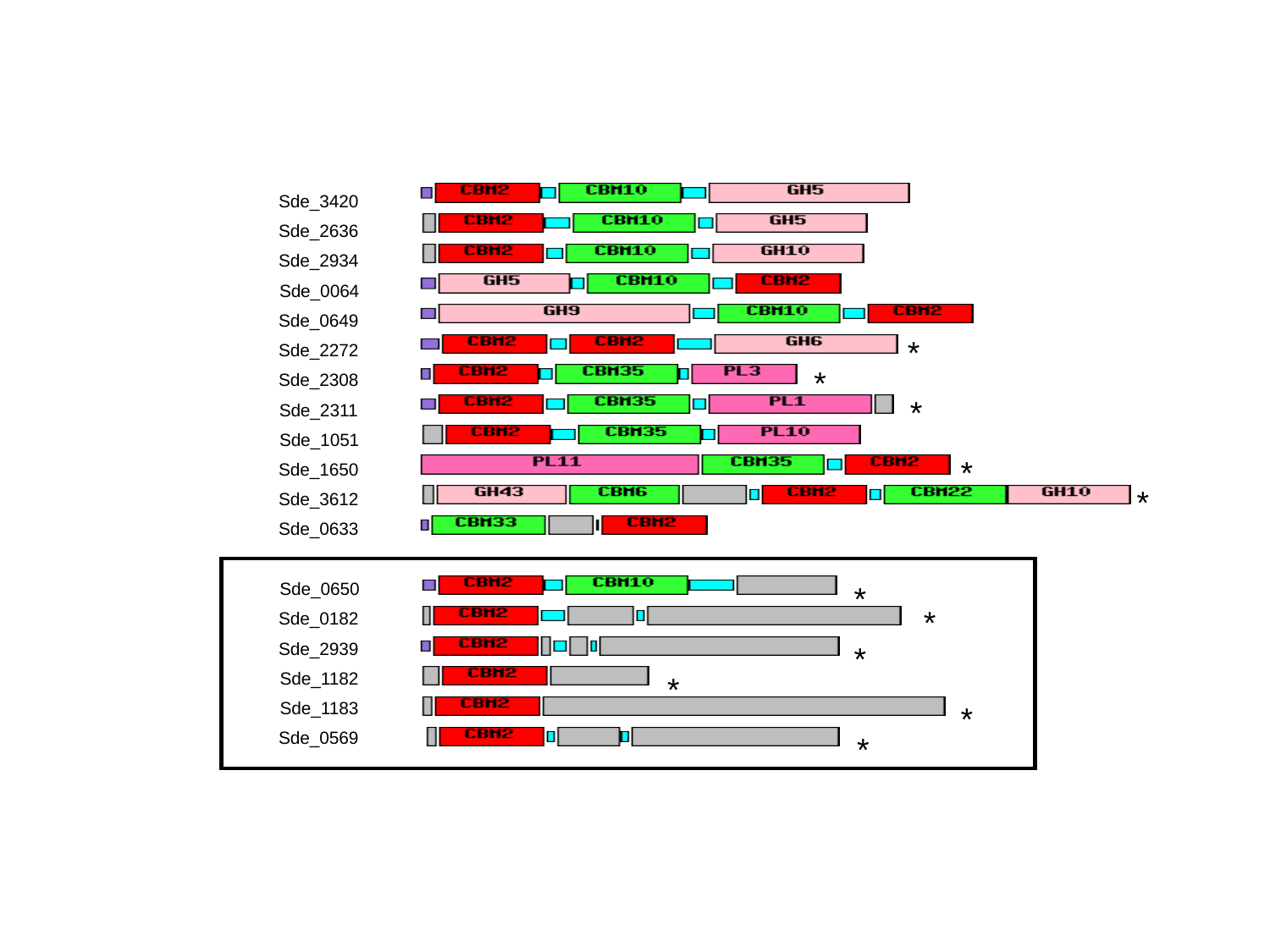

Sde_3420
Sde_2636
Sde_2934
Sde_0064
Sde_0649
*
Sde_2272
*
Sde_2308
*
Sde_2311
Sde_1051
*
Sde_1650
*
Sde_3612
Sde_0633
Sde_0650
*
*
Sde_0182
Sde_2939
*
Sde_1182
*
Sde_1183
*
Sde_0569
*

Supplement: Figure S1 — S. degradans proteins carrying CBM2 domains. Asterisks identify novel combinations of CBMs and catalytic domains. The boxed proteins have CBMs attached to domains of, as yet, completely unknown function. (0.07 MB PPT) [file pgen.1000087.s001.ppt]

## Slide 1
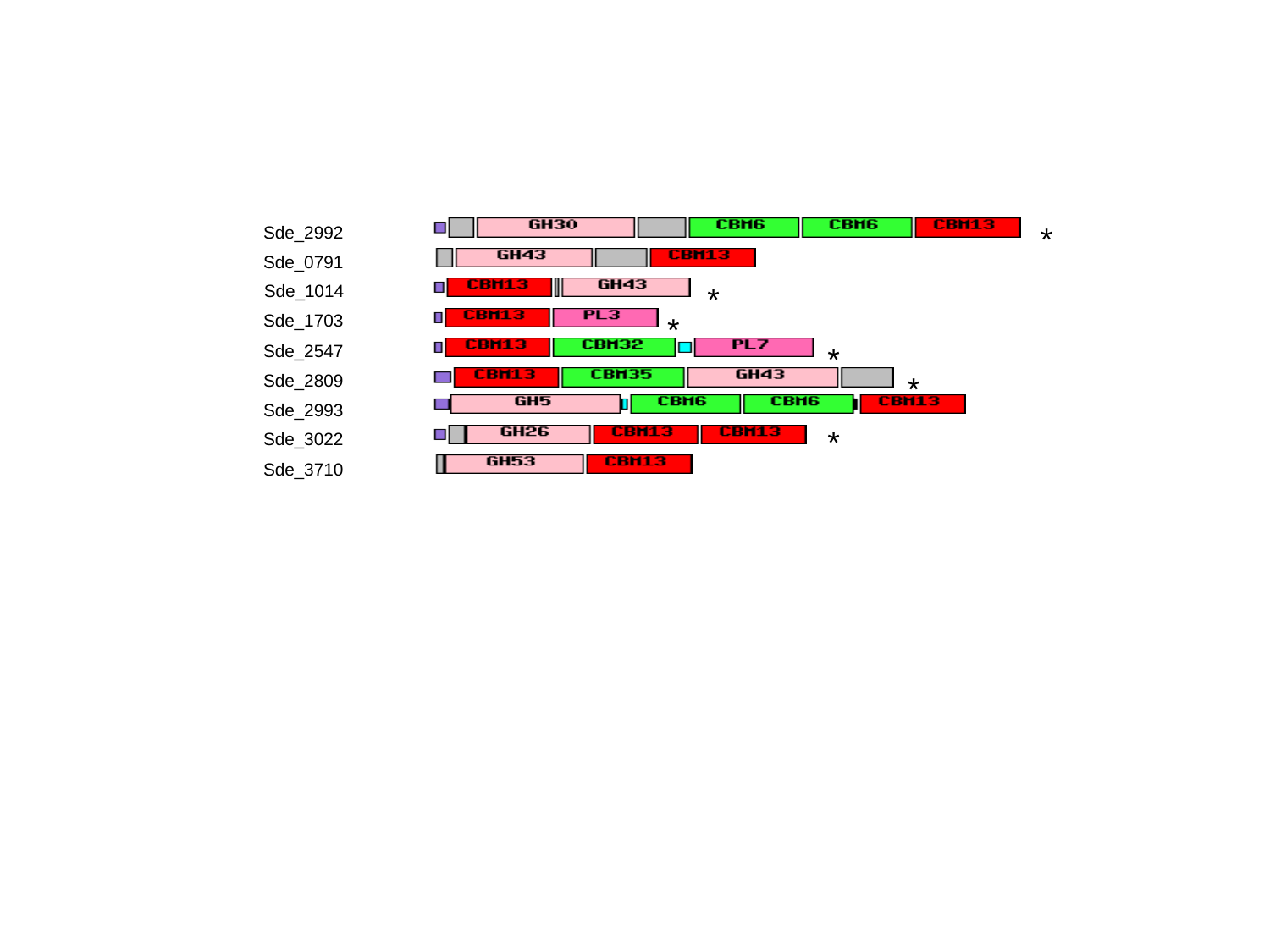

*
Sde_2992
Sde_0791
*
Sde_1014
*
Sde_1703
Sde_2547
*
Sde_2809
*
Sde_2993
*
Sde_3022
Sde_3710

Supplement: Figure S3 — S. degradans proteins carrying CBM13 domains. Asterisks identify novel combinations of CBMs and catalytic domains. (0.06 MB PPT) [file pgen.1000087.s003.ppt]

## Slide 1
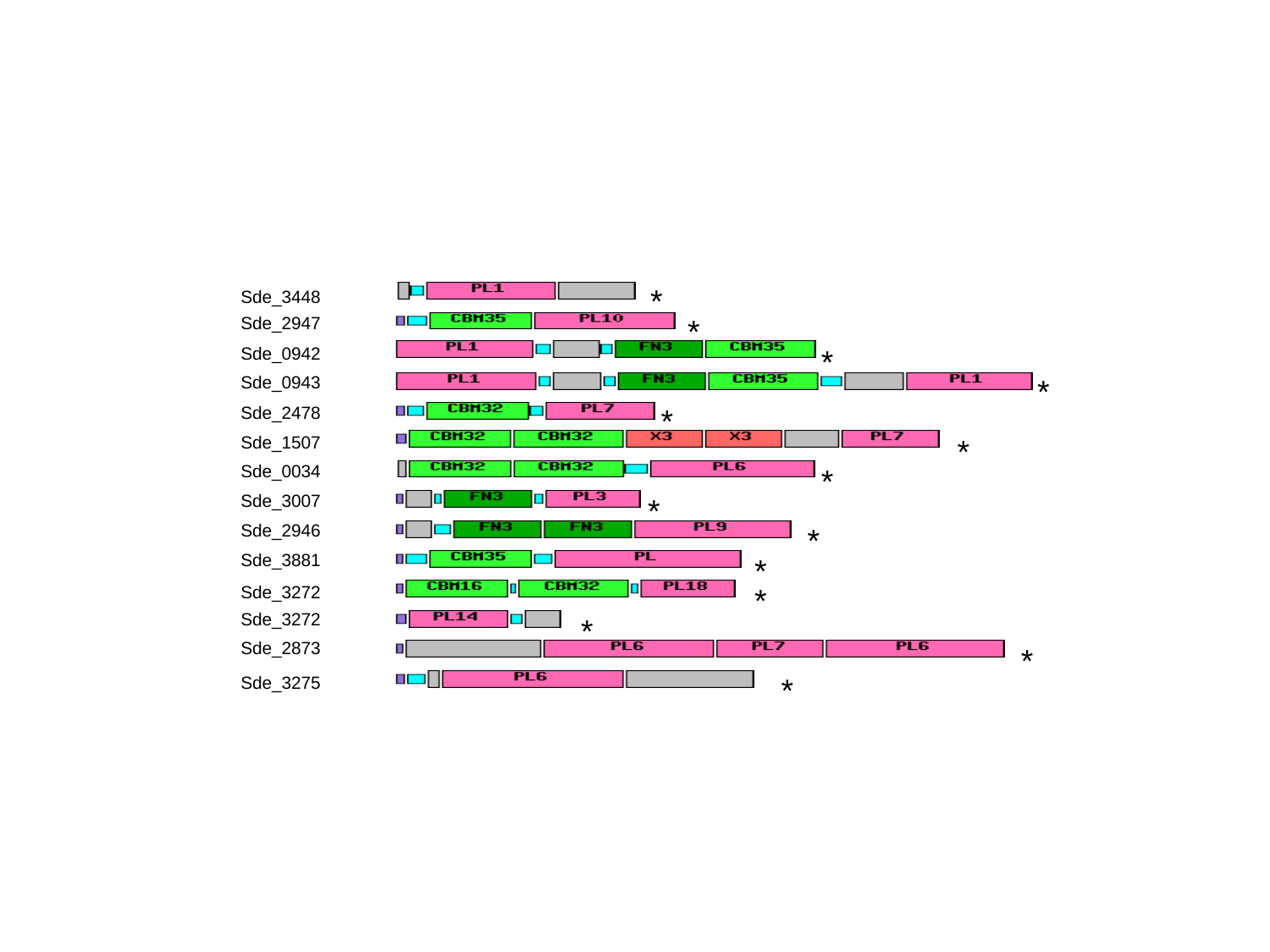

*
Sde_3448
*
Sde_2947
*
Sde_0942
Sde_0943
*
Sde_2478
*
Sde_1507
*
Sde_0034
*
Sde_3007
*
Sde_2946
*
Sde_3881
*
*
Sde_3272
Sde_3272
*
Sde_2873
*
*
Sde_3275

Supplement: Figure S4 — S. degradans modular polysaccharide lyases. 23 of the 33 Sde 2-40 PLs are modular, a higher proportion than observed in any organism thus far. Asterisks identify novel combinations of CBMs and catalytic domains. (0.06 MB PPT) [file pgen.1000087.s004.ppt]

## Slide 1
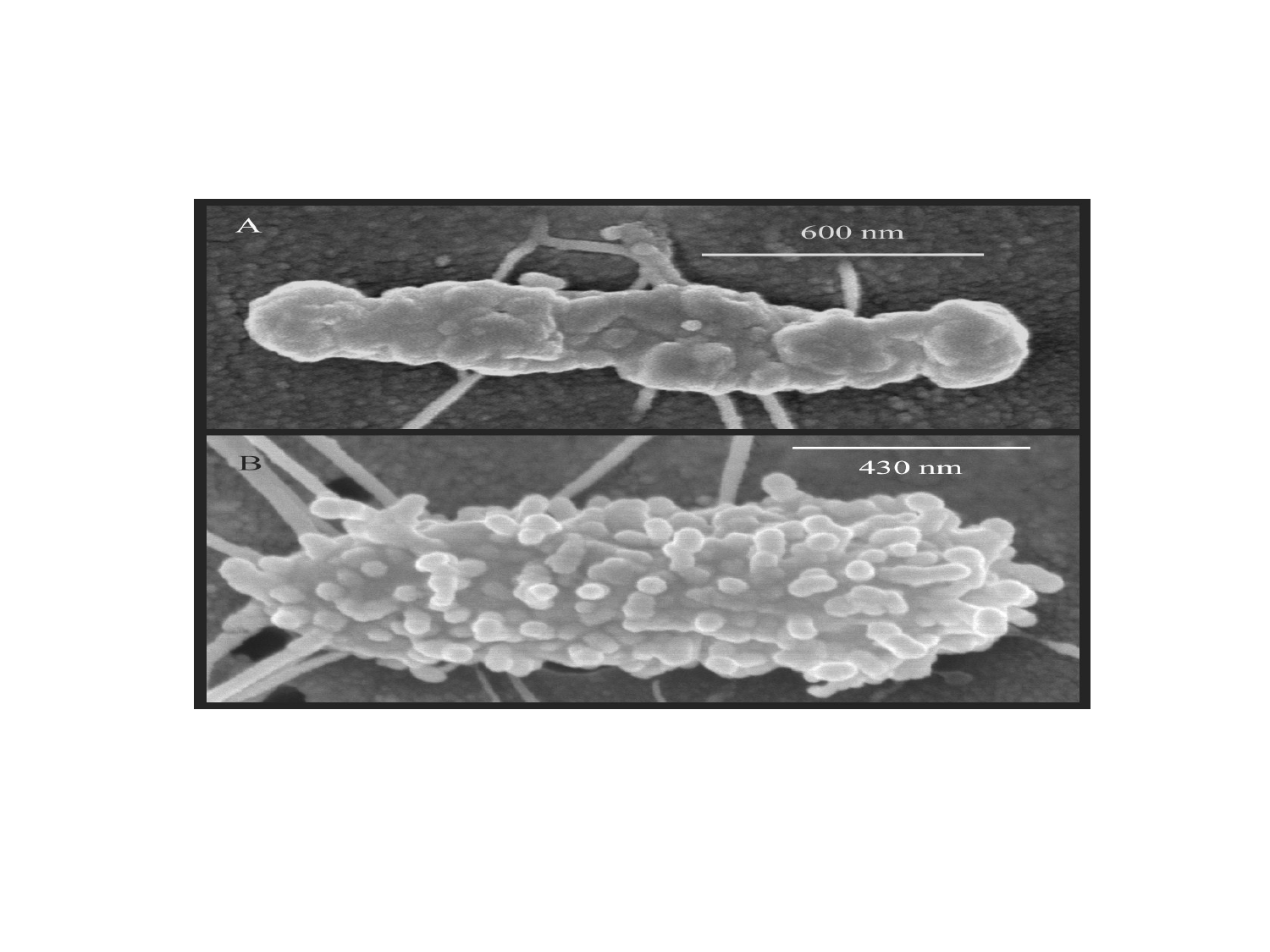

Supplement: Figure S5 — Scanning electron micrographs of S. degradans grown in minimal agarose medium. Cells were harvested at the indicated growth stage, washed twice and resuspended in 20 mM PIPES buffer, pH 6.8, amended to 1% final concentration glutaraldehyde and immobilized onto. 0.2 µm pore size Nucleopore 13 mm polycarbonate filters (Whatman, Middlesex, UK) followed by post-fixing in 2% (v/v) osmium tetraoxide (OsO4) and dehydration in a standard ethanol series. After critical point drying in CO2, the specimens were mounted and coated with ∼10 nm gold/palladium. Specimens were viewed on a Hitachi S-4700 ultra high resolution scanning electron microscope (UHR-SEM). A) Cell of S. degradans grown to mid-log phase exhibiting typical morphology and surface topology consisting of knobs at the polar termini and large, irregular surface protuberances. B) Late-stationary phase cell having typical shortened morphology and abundant smaller protuberances and apparent fibrilar appendages. (1.02 MB PPT) [file pgen.1000087.s005.ppt]
