# Supplementary material for: Complete Genome Sequence of the Complex Carbohydrate-Degrading Marine Bacterium, Saccharophagus degradans Strain 2-40T
Source: PLoS Genet. 2008 May 30;4(5):e1000087. doi: 10.1371/journal.pgen.1000087 (PMC2386152; doi:10.1371/journal.pgen.1000087)
Supplement: Figure S2 — S. degradans proteins carrying CBM6 domains. Asterisks identify novel combinations of CBMs and catalytic domains. The boxed proteins have CBMs attached to domains of, as yet, completely unknown function. (0.07 MB PPT) [file pgen.1000087.s002.ppt]

## Slide 1
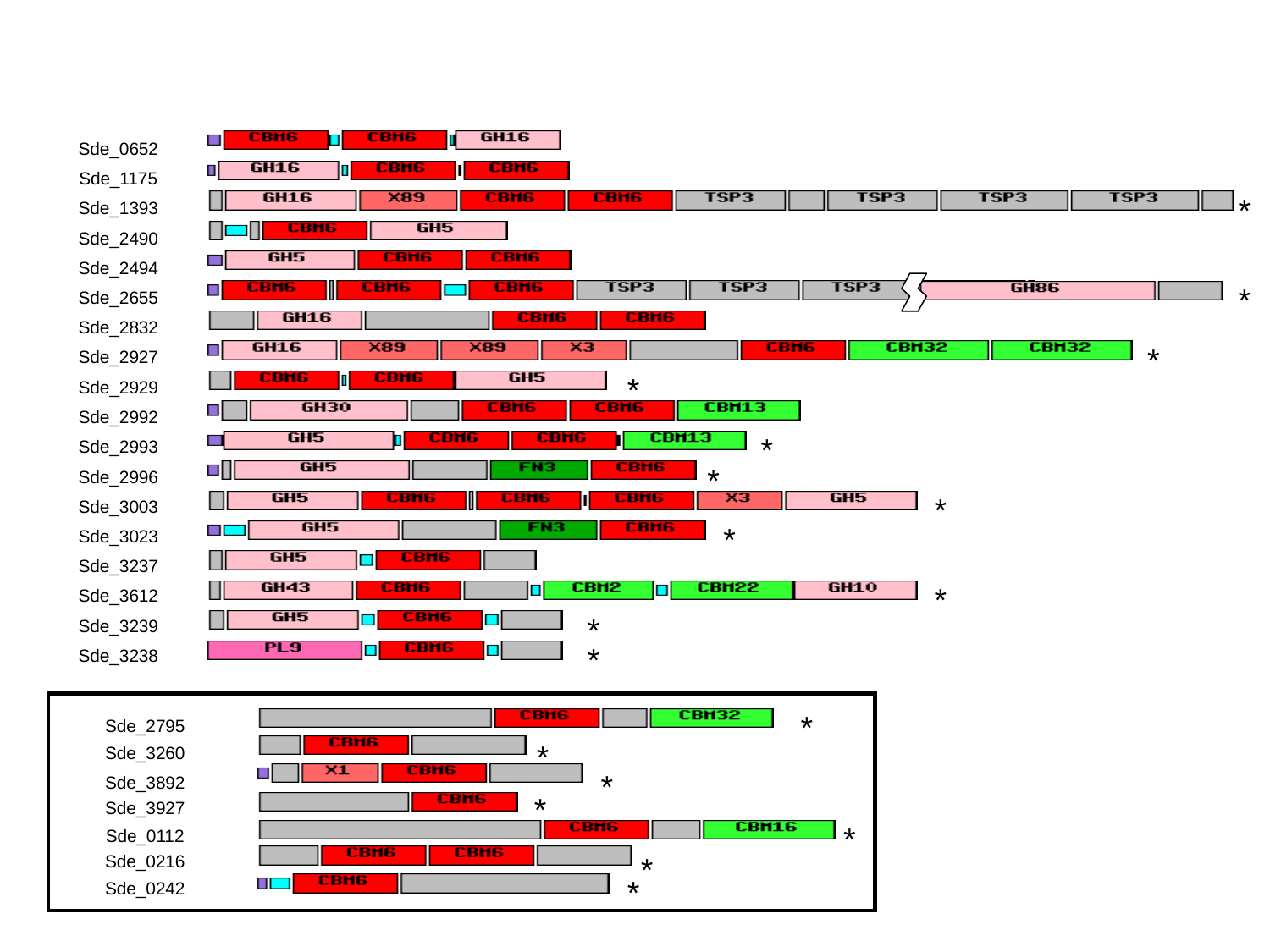

Sde_0652
Sde_1175
*
Sde_1393
Sde_2490
Sde_2494
*
Sde_2655
Sde_2832
*
Sde_2927
*
Sde_2929
Sde_2992
*
Sde_2993
*
Sde_2996
*
Sde_3003
*
Sde_3023
Sde_3237
*
Sde_3612
*
Sde_3239
*
Sde_3238
*
Sde_2795
*
Sde_3260
*
Sde_3892
*
Sde_3927
*
Sde_0112
*
Sde_0216
*
Sde_0242
