# Supplementary material for: Complete Genome Sequence of the Complex Carbohydrate-Degrading Marine Bacterium, Saccharophagus degradans Strain 2-40T
Source: PLoS Genet. 2008 May 30;4(5):e1000087. doi: 10.1371/journal.pgen.1000087 (PMC2386152; doi:10.1371/journal.pgen.1000087)
Supplement: Table S1 — Frequency of COG's in S. degradans 2-40. (0.05 MB DOC) [file pgen.1000087.s006.doc]

**Table S1.** Frequency of COG’s in *S. degradans* 2-40 (Sde 2-40)

| Description | COGs^1^ | Sde 2-40^2^ | Gamma^3^ | Bacteria^4^ |
| --- | --- | --- | --- | --- |
| Replication, recombination and repair | 123 | 2.7778 | 5.1961 | 4.4295 |
| Cell cycle control | 34 | 0.7678 | 0.6737 | 0.6910 |
| Transcription | 194 | 4.3812 | 4.8981 | 4.9477 |
| RNA processing and modification | 1 | 0.0226 | 0.0242 | 0.0114 |
| Translation | 154 | 3.4779 | 3.3678 | 3.6565 |
| Posttranslational modification, protein turnover, chaperones | 138 | 3.1165 | 2.8264 | 2.6152 |
| Defense mechanisms | 38 | 0.8582 | 0.9882 | 1.1522 |
| Signal transduction mechanisms | 252 | 5.6911 | 3.6227 | 3.4483 |
| Cell wall/membrane biogenesis | 184 | 4.1554 | 4.3054 | 4.1094 |
| Cell motility | 108 | 2.4390 | 2.0685 | 1.3776 |
| Cytoskeleton | 1 | 0.0226 | 0.0057 | 0.0052 |
| Intracellular trafficking and secretion | 107 | 2.4164 | 2.2961 | 1.7173 |
| Energy production and conversion | 146 | 3.2972 | 4.3095 | 4.3745 |
| Carbohydrate transport and metabolism | 180 | 4.0650 | 3.5918 | 3.4880 |
| Amino acid transport and metabolism | 176 | 3.9747 | 5.9370 | 5.9060 |
| Nucleotide transport and metabolism | 45 | 1.0163 | 1.4513 | 1.6335 |
| Coenzyme transport and metabolism | 104 | 2.3487 | 2.6556 | 2.6540 |
| Lipid transport and metabolism | 95 | 2.1454 | 2.3512 | 2.6600 |
| Inorganic ion transport and metabolism | 150 | 3.3875 | 3.6514 | 3.6268 |
| Secondary metabolites biosynthesis, transport and catabolism | 72 | 1.6260 | 1.6157 | 1.7937 |
| General function prediction only | 335 | 7.5655 | 7.8357 | 8.1934 |
| Function unknown | 223 | 5.0361 | 4.7426 | 4.8112 |
| Not in COGs | 1568 | 35.4110 | 31.5744 | 32.6864 |
| All predicted proteins^5^ | 4008 |  |  |  |

^1^ A number of clusters of orthologous groups (COGs) related to the described category.

^2^ Percent of the Sde 2-40 sequence coding for COG.

^3^ Average percent of known sequence in the Gamma proteobacteria coding for COG.

^4^ Average percent of known sequence in the kingdom, Bacteria, coding for COG.

^5^ Without those encoded by pseudogenes.
