# Supplementary material for: Complete Genome Sequence of the Complex Carbohydrate-Degrading Marine Bacterium, Saccharophagus degradans Strain 2-40T
Source: PLoS Genet. 2008 May 30;4(5):e1000087. doi: 10.1371/journal.pgen.1000087 (PMC2386152; doi:10.1371/journal.pgen.1000087)
Supplement: Table S2 — Homologs of protein secretion system components. (0.07 MB DOC) [file pgen.1000087.s007.doc]

**Table S2. Homologs of protein secretion system components**

| Homolog | Locus Tag | Address |
| --- | --- | --- |
| Sec System |  |  |
| SecA | 0856 | 1111093 |
| SecB | 0497 | 609463 |
| SecD | 1407 | 1823239 |
| SecF | 1408 | 1825152 |
| SecE | 0918 | 1184003 |
| SecG | 2716 | 3443786 |
| SecY | 0979 | 1253932 |
| YajC | 1662 | 2130225 |
| YidC | 4014 | 5054707 |
| Signal peptidase I | 0576 | 713185 |
| Signal peptidase II | 2565 | 3254190 |
| Signal peptide peptidase | 897 | 1160074 |
|  |  |  |
| SRP System |  |  |
| *ffs* | R0021 | 1729370 |
| SRP54 M domain | 1202 | 1547130 |
| SRP54 G domain | 2166 | 2758897 |
| FtsY | 3597 | 4556332 |
|  |  |  |
| Tat System |  |  |
| TatA/E | 3224 | 4099571 |
| TatB | 3225 | 4099825 |
| TatC | 3226 | 4100324 |
|  |  |  |
| Type I Secretion |  |  |
| HlyB | 1291 | 1654333 |
| HlyD | 1292 | 1656453 |
| TolC | 1293 | 1658124 |
|  |  |  |
| Type II Secretion |  |  |
| Cluster 1 |  |  |
| GspC | 3581 | 4541255 |
| GspD | 3580 | 4539252 |
| GspE | 3579 | 4537735 |
| GspF | 3578 | 4536478 |
| GspG | 3577 | 4535967 |
| GspH | 3576 | 4535379 |
| GspI | 3575 | 4534966 |
| GspJ | 3574 | 4534335 |
| GspK | 3573 | 4533269 |
| GspL | 3572 | 4532020 |
| GspM | 3571 | 4531535 |
| GspN | 3570 | 4530745 |
| GspO | 0861 | 1116889 |
|  |  |  |
| Cluster 2 |  |  |
| GspD | 1135 | 1469959 |
| GspE | 1138 | 1473946 |
| GspF | 1139 | 1475694 |
| GspG | 1140 | 1476917 |
| GspG | 1141 | 1477589 |
| GspG | 1142 | 1478002 |
| GspG | 1144 | 1478522 |
|  |  |  |
| Cluster 3 |  |  |
| GspE | 1345 | 1740751 |
| GspF | 1346 | 1742450 |
| GspH | 1351 | 1747531 |
